# Alcohol Intake and Serum Glucose Levels from the Perspective of a Mendelian Randomization Design: The KCPS-II Biobank

**DOI:** 10.1371/journal.pone.0162930

**Published:** 2016-09-15

**Authors:** Yon Ho Jee, Sun Ju Lee, Keum Ji Jung, Sun Ha Jee

**Affiliations:** 1 Institute for Health Promotion, Graduate School of Public Health, Yonsei University, Seoul, Korea; 2 Department of Epidemiology and Health Promotion, Graduate School of Public Health, Yonsei University, Seoul, Korea; Shanghai Diabetes Institute, CHINA

## Abstract

**Background:**

Previous studies have suggested that alcohol intake is associated with increased fasting serum glucose (FSG), but the nature of the relationship remains unknown. We used Mendelian randomization analysis to assess the causal effect of alcohol intake on FSG in a middle-aged Korean population.

**Methods:**

Clinical data including FSG and alcohol intake were collected from 156,386 Koreans aged 20 years or older who took part in the Korean Cancer Prevention Study-II (KCPS-II) Biobank Cohort. The single nucleotide polymorphism rs671 in ALDH2 was genotyped among 2,993 men and 1,374 women in 2016. This was a randomly selected subcohort of KCPS-II Biobank participants.

**Results:**

Alcohol consumption was positively associated with FSG level in men, but not in women. The rs671 major G allele was associated with increased alcohol intake (F-statistic = 302.62) and an increase in FSG in men. Using Mendelian randomization analysis, alcohol intake increased FSG by 1.78 mg/dL per alcohol unit (10 g ethanol) per day (95% CI: 0.97–2.59) in men. The associations became stronger when we excluded heavy drinkers and the elderly. However, in women, no significant association between rs671 and alcohol or serum glucose was found.

**Conclusion:**

Using Mendelian randomization analysis, we suggest a causal relationship between alcohol intake and FSG among Korean men. Moreover, we found that the ALDH2 variant rs671 was not associated with FSG among Korean women.

## Introduction

Diabetes mellitus is a common, serious, and costly disease in many countries, including Korea. Approximately 150 million individuals worldwide have diabetes mellitus, and due to factors such as population growth, aging, alcohol consumption, obesity, and sedentary lifestyles, the number, especially in developing countries, may double by 2025 (WHO, 2016) [1]. The association between alcohol consumption and fasting serum glucose level (FSG) is inconsistent in epidemiologic studies [2, 3]. A systematic review of five prospective cohort studies in Japan reported that moderate alcohol consumption was associated with an increased risk of type 2 diabetes in subjects with a low body mass index (BMI) [2]. In contrast, a recent meta-analysis on the association between alcohol consumption and the risk of diabetes reported that light-to-moderate alcohol consumption was associated with a lower risk of type 2 diabetes (T2D), whereas heavy alcohol consumption was not related to the risk of T2D (Li XH 2016) [4]. However, the role of alcohol consumption as a risk factor for FSG is still uncertain due to potential confounding factors. Given the limitations of the observational studies, whether the association of alcohol intake with FSG is causal or is due to residual confounding remains to be determined.

Mendelian randomization (MR) rests on the assumption that genetic variants assigned prior to conception are randomly allocated according to Mendel’s second law [5, 6]. As instrumental variables for MR analysis, studies on genetic polymorphisms in major alcohol-metabolizing enzyme genes such as alcohol dehydrogenases (ADH), aldehyde dehydrogenase (ALDH), and cytochrome P450 2E1 (CYP2E1) have been reported [7]. In particular, the rs671 variant of ALDH2 is well known as a critical region related to alcohol drinking behavior [8, 9].

The causal effects of alcohol on health can be determined using genetic markers and aldehyde dehydrogenase 2 (ALDH2) with single nucleotide polymorphisms (SNPs) for instrumental variable analysis [10]. The inactivated ALDH2 alleles slow down the metabolism of acetaldehyde and cause upset stomach after alcohol consumption [11].

Using the Korean Cancer Prevention Study-II (KCPS-II) Biobank Cohort, we hypothesized that higher levels of alcohol intake would increase FSG with the use of alcohol and the ALDH2 gene variants. Taking advantage of the unique Korean setting, in which alcohol intake is generally low-to-moderate and of which may reflect more genetic variation in men, we used ALHD2 as a genetic instrument to obtain an unbiased estimate of the effect of alcohol intake on FSG.

## Materials and Methods

### Participants

The Korean Cancer Prevention Study-II (KCPS-II Biobank) was initiated in April 2004 and was supported by the Seoul city government in December 2005 as a part of the Korean Metabolic Syndrome Research Initiative study. The blood samples collected from the KCPS-II Biobank were initially taken in 2004, from two hospitals (Severance Hospital, Bundang Cha Hospital) and expanded to eleven hospitals (Korean Medical Institute, Ewha Womans University Mokdong Hospital, Seoul National University Hospital, Korea University Hospital, Kyung Hee University Medical Center, The Catholic University of Korea Buchun ST. Marys Hospital, Seoul Medical Center, Asan Medical Center, and Hanyang University Guri hospitals) since April 2006. These eleven health promotion centers located in Seoul and Kyong-gi province consist of 18,879,351 people (about 40.9% out of the total South Korean population of 46,136,101) based on the 2000 report (KOSIS) [12]. In fact, the population density in Seoul and Kyong-gi was 16,342.2 persons/km^2^ and 886.4 persons/km^2^, respectively, in 2000. Study subjects received routine health assessments at health promotion centers across South Korea. The number of participants who had provided informed consent in eleven health promotion centers between 2004 and 2013 was 159,836. The final number of study subjects was 5,000, which was a randomly selected subcohort of KCPS-II Biobank participants.

The health examinations included questions on lifestyle, family, personal medical history, and assessment of anthropometric and clinical factors. General information on socioeconomic positions and lifestyle, including age, sex, education level, smoking status, and alcohol intake were collected by a standardized questionnaire. Alcohol intake was recorded in terms of frequency, type of beverage, and usual amount consumed per occasion.

We excluded the following numbers of participants because of missing information: 496 who had no data on alcohol consumption amount, 95 who had medication history regarding diabetes, 31 who had no information of rs671 genotyping and 11 participants with extremely high alcohol amount (≥ 200g alcohol unit/day) were excluded. The final study subjects are 4,367 individuals.

The Severance Medical Ethics Committee approved the study (no. 4-2011-0277), and all participants provided written, informed consent prior to participation.

### DNA extraction and SNP analysis

The biological samples for DNA extraction used in the present study were obtained from the KCPS-II Biobank at baseline. ALDH2 genotyping was conducted in 2016, using a randomly selected subcohort of KCPS-II Biobank. Genotype data were produced using the Korean Chip (K—CHIP) obtained from the K—CHIP consortium.

### Hybridization on Affymetrix Axiom™ KORV1.0–96 Array

According to manufacturer’s protocol, it is recommended to use Axiom®2.0 Reagent Kit (Affymetrix Axiom®2.0 Assay User Guide). About 200ng of genomic DNA is amplified and randomly fragmented into 25 to 125 base pair (bp) fragments. gDNA initial amplification reacted in 40㎕ reaction volume, containing 20㎕ volume of genomic DNA at a concentration of 10ng/㎕, 20㎕ of Denaturation Master Mix. The reaction of initial amplification carried out as follows: 10min at Room Temperature. After the initial amplification, the incubated products were amplicated with 130㎕ of Axiom 2.0 Neutral Soln, 225㎕ Axiom 2.0 Amp Soln and 5㎕ Axiom 2.0 Amp Enzyme. The amplification reactions were carried out as follows: 23hour ± 1hour at 37°C. The amplification products were performed in optimized reaction to amplify fragments between 200–1,100 base pairs. A fragmentation step then reduced the amplified products to segments of approximately 25–50 bp, which were then end-labeled using biotinylated nucleotides. Following hybridization, the bound target is washed under stringent conditions to remove non-specific background to minimize background noise caused by random ligation events. Each polymorphic nucleotide is queried via a multi-color ligation event carried out on the array surface. After ligation, the arrays are stained and imaged on the GeneTitan MC Instrument (Affymetrix, Santa Clara, CA, USA). The image was analyzed using Genotyping Console™ Software (Affymetrix, Santa Clara, CA, USA). Genotype data were produced using the K-CHIP available through the K-CHIP consortium. K-CHIP was designed by Center for Genome Science, Korea National Institute of Health, Korea (4845–301, 3000–3031).

### Alcohol intake

Amount of alcohol intake was assessed as a number of alcohol units (10 gram (g) ethanol) per day, obtained from the frequency, quantity, and type of drinking recorded at baseline. All participants were asked how often they drank alcohol (0 to 7 days per week), the type of alcohol usually consumed, and amount of each alcohol (beer, Western grape wine, spirits, Korean rice wine, or the Korean spirit Soju (high strength)) they usually consumed per occasion, from which we calculated units per day. Participants who reported drinking >200g alcohol units per day were excluded from the study [11]. “Never drinkers” were those who never drank alcohol.

### Outcome

Serum FSG concentrations were the primary outcome. FSG was measured with a COBAS INTEGRA 800 and a 7600 Analyzer (Hitachi, Tokyo, Japan). The FSG measurement followed internal and external quality control procedures, as required by the Korean Association of Laboratory Quality Control. Agreement for FSG across individual hospital was high (correlation coefficient range 0.96–0.99) [13].

### Statistical analysis

We performed analyses in three steps. First, linear regression was used to assess the strength of the association of ALDH2 variants (rs671) with alcohol units. The strength of the association is expressed by the F statistic. We used linear regression under the assumption of an additive genetic model. Second, we examined the association of rs671 with participant characteristics, including age, sex, education, smoking, physical activity, and family history of diabetes. Third, we used linear regression to assess the association of rs671 with FSG. We estimated the effect size for FSG per each copy increase in the rs671 (G) allele. A two-sided significance level of α = 0.05 was used. All statistical analysis was done using SAS 9.2 (SAS Institute Inc, Cary, NC, USA) and STATA/IC 13.1 (Stata Corp LP, College Station, TX, USA).

## Results

A total of 2,993 men and 1,374 women from the KCPS-II Biobank, and measurements of their FSG levels and ALDH2 genotypes, were included in the analysis. 89.4% of men and 44.9% of women were alcohol users.

Table 1 shows that ALDH2 in men was strongly associated with alcohol intake, such that men with two G alleles, which confers fast acetaldehyde metabolism, consumed 1.6 units of alcohol per day compared with those with slow acetaldehyde metabolism (0.3 units) (P < .0001). Also, male participants with GG genotype had increased FSG levels (91.7 mg/dL) compared those with AA genotype (87.9 mg/dL) (P < .0001). However, these associations were not significant in women, most likely because of the lower prevalence of alcohol consumption. Rs671 was not associated with age, education, smoking, physical activity, or family history of diabetes in both men and women.

**Table 1:** Alcohol consumption and general characteristics by ALDH2 polymorphism at rs671 in men and women from the KCPS-II Biobank.

		ALDH2 polymorphism rs671			
		AA	GA	GG	P value‡
**Men** (n = 2,993)		76 (2.5%)	819 (27.4%)	2,098 (70.1%)	
Alcohol units, 10g ethanol †	Geometric mean (SD)	0.3 (0.7)	0.7 (1.1)	1.6 (1.1)	< .0001
Fasting serum glucose, mg/dL	Mean (SD)	87.9 (13.8)	89.0 (15.9)	91.7 (16.5)	< .0001
Age, year	Mean (SD)	42.2 (9.4)	42.5 (8.6)	41.9 (8.6)	0.2225
Education, %*	High school	13.8	16.0	20.3	0.2030
	College or above	82.8	76.8	75.5	
Smoking status. %	Former	23.7	25.4	28.6	0.3478
	Current	46.1	48.1	44.7	
Ever alcohol drinking, %	Yes	23.7	80.2	95.3	< .0001
Physical activity, %	Active	65.8	64.8	67.3	0.4418
Family history of diabetes, %	Yes	14.5	16.8	17.1	0.7972
**Women** (n = 1,374)		33	356	985	
Alcohol units, 10g ethanol	Geometric mean (SD)	0.0 (0.0)	0.3 (0.8)	0.4 (1.0)	< .0001
Fasting serum glucose, mg/dL	Mean (SD)	86.3 (13.6)	86.5 (12.9)	86.4 (11.8)	0.9824
Age, year	Mean (SD)	41.3 (6.3)	43.6 (10.1)	42.6 (9.7)	0.2483
Ever alcohol drinking, %	Yes	0.0	20.8	55.1	< .0001
Education, %	High school	55.6	33.8	33.3	0.1127
	College or above	38.9	45.2	51.5	
Smoking status. %	Former	9.1	5.6	3.5	0.1785
	Current	0.0	3.7	3.6	
Physical activity, %	Active	45.5	50.8	53.4	0.5045
Family history of diabetes, %		18.1	16.0	15.3	0.8750

†10 g ethanol per day

‡P-value from analysis of variance (ANOVA) for continuous variables and from χ2 test for categorical variables, 2 sided. Alcohol units were log transformed before the ANOVA analysis.

*Two thousand eight hundred thirty-one missing

The F-statistic for the association of rs671 with alcohol intake in men was significantly higher than in women (302.62 in men; 44.30 in women). Rs 671 is not an instrumental variable for alcohol in women. The F-statistics became even stronger when we excluded heavy drinkers (alcohol ≥100 g/day) (range of F statistics: 301.85 to 327.10) (Table 2). In men, alcohol intake increased FSG in instrumental variable analysis (2.51 mg/dL per alcohol unit, 95% CI 0.98–2.58) when we excluded heavy drinkers (alcohol intake ≥50 g/day). A much lower association was shown in observational multivariate regression, with a positive association between alcohol and FSG in men (0.52 mg/dL per alcohol unit, 95% CI 0.28–0.76) (Table 2).

**Table 2:** Association of alcohol (10g ethanol) with fasting serum glucose using Mendelian Randomization analysis.

			Observational multivariable regression analysis* X-Y		Mendelian randomization analysis			
	Selection	Number	β	SE	F-statistic G-X	P for endogenous test	β	SE
Total	Total*	4,367	0.49	0.11	280.94	0.0344	1.57	0.43
	Excluding heavy users ^1)^	4,306	0.70	0.14	327.10	0.0773	1.75	0.48
	Excluding heavy users ^2)^	4,059	0.54	0.20	301.85	0.0518	2.11	0.71
	Excluding elderly ^3)^	4,262	0.51	0.11	275.49	0.0280	1.64	0.43
Men	Total*	2,993	0.52	0.12	302.62	0.0009	1.78	0.41
	Excluding heavy users^1)^	2,932	0.76	0.16	370.80	0.0026	2.01	0.47
	Excluding heavy users^2)^	2,699	0.63	0.23	371.60	0.0004	2.51	0.67
	Excluding elderly^3)^	2,936	0.54	0.12	296.18	< .0001	1.78	0.41
Women	Total*	1,374	0.32	0.05	44.30	0.8844	-0.21	2.02
	Excluding heavy users^1)^	1,374	0.32	0.05	44.30	0.8844	-0.21	2.02
	Excluding heavy users^2)^	1,360	0.24	0.03	55.12	0.7514	-0.30	2.71
	Excluding elderly^3)^	1,326	0.32	0.05	43.76	0.6245	0.53	1.98

*Adjusted for age, sex, smoking status, exercise, and family history of diabetes

1) Participants with alcohol intake <100 g/day

2) participants with alcohol intake <50 g/day

3) Participants aged 65 years or older

Tests of endogeneity is Wu-Hausman test, Ho: variables are exogenous

**Fig 1:**
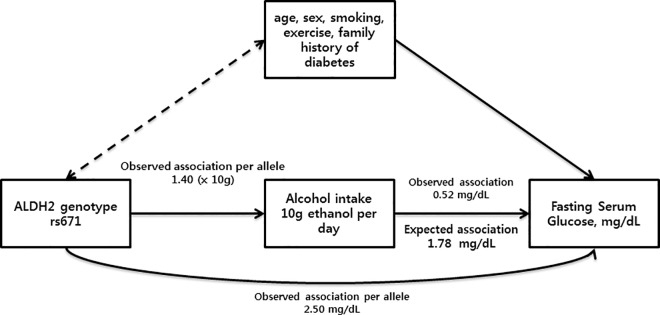
Mendelian randomization of alcohol intake and fasting serum glucose in men: the KCPS-II Biobank.

Fig 1 showed a triangulation approach used to compare the observed association and expected association. The observed effect of genotype on alcohol intake (β genotype-alcohol intake) and risk of an increased FSG (β genotype-FSG) was estimated per each copy of the G allele of rs671. The observed effect of alcohol intake on risk of an increased FSG was estimated per 10 g ethanol per day. The estimated causal risk of an increased FSG was 1.78 mg/dL.

In women, the F-statistic for the association of rs671 with alcohol intake was 44.30, and the p-value for endogenous test was 0.8844, indicating that rs671 does not use an instrument for alcohol intake in women. In this setting, alcohol intake was negatively correlated with rs671. However, the two were positively correlated when we excluded elderly individuals. Neither association was statistically significant.

## Discussion

The Korean population, relative to their Western counterparts, is notable for its low frequency of obesity but high alcohol intake. We found that higher alcohol intake was associated with higher FSG using Mendelian randomization analysis and that alcohol intake may reflect genetic differences in men. However, alcohol intake was not associated with FSG among women. A recent meta-analysis on the association between alcohol consumption and the risk of diabetes reported that light and moderate alcohol consumption was associated with a lower risk of T2D, whereas heavy alcohol consumption was not related to the risk of T2D [11].

In our Mendelian randomization study, the association became stronger when we excluded heavy drinkers (≥100 g/day or ≥50 g/day alcohol). The stronger association in Mendelian randomization analysis after excluding heavy users could be due to canalization and developmental adaptation [11, 14]. The genetic effect of alcohol on FSG may be modified via compensatory responses to environmental influences, i.e. upregulation of ALDH2 gene expression, to protect heavy alcohol users from increased FSG. This would also attenuate the association of genetically determined alcohol intake with FSG.

The association in our study was stronger than that found in the Guangzhou Biobank cohort study in Chia. The difference between these two studies may be because of the difference in G allele frequency of rs671. The prevalence of the G and GG genotypes in men in our study were 27.4% and 70.1%, respectively, while the corresponding rates in the Guangzhou Biobank cohort study were 41.8% and 48.7%, respectively. The frequencies of the G allele were similar in men and women. Of course, samples from both studies may not be representative of the populations in Korea and China. Therefore, we need to carefully interpret the results while considering genetic differences.

Meanwhile, rs671 was also reported to be associated with visceral fat accumulation in the East Asians in China [15]. The results implied that alcohol consumption may mediate the impact of the ALDH2 locus on visceral fat in a Chinese population. Of course, visceral fat is well known as strong risk factor of an increased risk of diabetes. Therefore, our data also implied that alcohol consumption may mediate the impact of the ALDH2 locus on fasting glucose in a Korean population.

In present study, we found a different association in men than in women, perhaps because rs671 is not itself causal, or the association is more complex than is expressed only in rs671. However, it has been reported that rs671, located in exon 12 of the ALDH2 locus, directly causes the alcohol consumption, primarily in men. The study suggests that no casual association in women may be due to the social pressure for Asian women to abstain from alcohol drinking. Further studies are needed to conclude this part.

In our study, ALDH2 has shown to be an adequate and validated instrument (F statistic 280.94) for alcohol intake in Koreans. The F statistic was slightly greater in men (F statistic 302.62). Likewise, ALDH2 was a strong instrumental variable in the current Mendelian randomization study. The genetic effect of a one unit change in alcohol intake on FSG in men was four times larger than the association seen in our observational study. However, among women, in whom alcohol intake is less likely a reflection of genetic differences because of a relatively low cultural acceptance of alcohol intake [11], these same genetic variants were not associated with FSG. Our results in women are similar to those in the above-mentioned study [11]. However, in our study, although not statistically significant, alcohol intake was positively associated with FSH when we excluded elderly people. These results suggest that individuals in the younger generation are less likely constricted by social norms. A further study on the younger generation should be conducted to confirm this hypothesis. In 2015, Xu et al. reported results similar to ours, despite of the differences observed in G allele frequencies of rs671. They provided several possible explanations [11]. First, the Mendelian randomization study design incurs less bias from both measured and unmeasured confounding variables. The observational study design is more likely to have confounding bias, which distorts the association and results in null association. Second, the Mendelian randomization study assumed that alcohol intake was assessed without random measurement error. Although, alcohol intake is a very strong instrumental variable in our Mendelian randomization study, alcohol intake still has measurement error. Alcohol amount is notoriously difficult to measure accurately [16].

Given the limited experimental data on alcohol and FSG in Korea, further studies should be done to understand whether the effect of alcohol on FSG seen here using Mendelian randomization differs from what would be expected from an intervention. Nevertheless, our findings suggest that the effect of alcohol on FSG is substantially larger than that usually reported.

To the best of our knowledge, only one Mendelian randomization analysis has examined the causal effect of alcohol intake on FSG in Korea, and we did not perform a replication analysis in a second group. Therefore, more cohorts are needed to verify these results in other populations and ethnicities, which will be of a great interest in the future. Another limitation of our study is potential existence of pleiotropy [17].

All Mendelian randomization studies are confounded by linkage disequilibrium. A previous group evaluated genetic instruments for alcohol intake in China and concluded that ALDH2 variants are a credible genetic instrument for alcohol intake in Mendelian randomization studies and many other health attributes in Southern Chinese men [10]. A similar study should be conducted in the near future in Korea. Third, ALDH2 mainly varies in East Asians. The allele frequencies of rs671 of ALDH2 differ among populations: the 504Lys allele (“A” allele) is common in East Asians, including Japanese, but quite rare in other populations of European and African ancestry [18]. The effects of alcohol on health may vary between East Asians, although this is unlikely. Confounding by population stratification cannot be completely ruled out.

However, a majority of the Korean population is ethnically homogenous, minimizing such negative effect [19]. Despite these limitations, the Mendelian randomization approach is a useful tool to assess the nature of the observed associations between putative risk factors and disease. This approach overcomes some potential limitations of observational studies, such as the presence of confounding variables [20].

## Conclusions

In conclusion, our study provides evidence for a causal effect of alcohol intake on FSG. Causal associations were stronger with light or moderate alcohol intake. Additional research regarding Mendelian randomization should be conducted and extended to a larger cohort, and individuals with prediabetes or diabetes should be investigated to illuminate the effect of alcohol intake on diabetes-related outcomes.
